# Opioid-Related Treatment Disparities Among Medicaid Enrollees in Indiana

**DOI:** 10.1089/heq.2021.0154

**Published:** 2023-02-01

**Authors:** Carolina Vivas-Valencia, Nan Kong, Nicole Adams, Paul M. Griffin

**Affiliations:** ^1^ Weldon School of Biomedical Engineering, Purdue University, West Lafayette, Indiana, USA.; ^2^ School of Nursing, Purdue University, West Lafayette, Indiana, USA.; ^3^ Department of Industrial and Manufacturing Engineering, Pennsylvania State University, University Park, USA.; ^4^ Consortium for Substance Use and Addiction, Social Science Research Institute, Pennsylvania State University, University Park, USA.

**Keywords:** opioid use disorder, treatment disparities, reimbursement claims data

## Abstract

**Introduction:**

Health care disparities based on race/ethnicity and sex can be found in a variety of settings. Our aim is to determine if there are disparities in treatment provided to Indiana Medicaid enrollees who have medically documented opioid use.

**Study Data and Methods:**

We used Medicaid reimbursement claims data to extract patients who were diagnosed with opioid use disorder (OUD) or had other medical event related to opioid use between January 2018 and March 2019. We used a two-proportion *Z*-test to verify the difference in the proportion of treatment provided between population subgroups. The study was approved by the Purdue University Institutional Review Board (2019–118).

**Study Results:**

Over the study period, there were 52,994 Indiana Medicaid enrollees diagnosed with OUD or documentation of another opioid related event. Only 5.41% of them received at least one type of treatment service (detoxification, psychosocial, medication assisted treatment, or comprehensive).

**Discussion:**

Although Medicaid began covering treatment services for enrollees with an OUD in Indiana at the start of 2018, very few received evidence-based services. Men and White enrollees with an OUD were in general more likely to receive services compared to women and non-White enrollees.

## Introduction

Opioid overdoses are a major public health crisis,^1^ and are related to opioid use disorder (OUD), which involves misuse of prescription opioid medications or illicitly obtained heroin and fentanyl. From the 2.4 million individuals estimated to have an OUD nationwide, roughly one in five receive any specialty care.^2^ Medications such as methadone, buprenorphine, and naltrexone, either administered as stand-alone therapies or in conjunction with behavioral therapy, have been found efficacious for treating OUD.^2^ Although efficacy and cost-effectiveness of several types of treatment have been established, individuals seeking treatment continue to encounter a fragmented system that creates barriers to effective services.^3^

For example, <10% of people with OUD receive medication-assisted treatment (MAT) nationwide,^4^ with a 6-month retention of <50% for MAT with methadone, buprenorphine, or naltrexone.^5^ Consequently, the combination of limited access and low retention rate in treatment programs can result in overdose deaths.^6^ In this brief, we examine the types of treatment provided to Indiana Medicaid enrollees who were diagnosed with an OUD between January 2018 and March 2019. We also determine whether race/ethnicity or sex were correlated to the receipt of treatment.

## Study Data and Methods

We used Medicaid reimbursement claims data obtained from the Indiana Family and Social Services Administration. We extracted patients who were diagnosed with an OUD between January 2018 and March 2019 (Supplementary Fig. S1) and enrolled in Medicaid for the entire duration. The time interval was partly chosen because the State of Indiana initiated the 1115 SUD Waiver Implementation Plan, which provided full coverage of opioid treatment program services to Medicaid eligible in Indiana, including daily administration of methadone.^7^

A total of 1.88 million people were covered by Indiana Medicaid for the study period. From this population, we also extracted patients diagnosed with problematic use of opioids, that is, those diagnosed with opioid abuse, opioid use, opioid dependence, and/or patients who had an emergency room visit or hospitalization with an opioid-related poisoning within the study period (ICD-10 diagnosis codes F11.XXX and T40.XXXX). We excluded codes F11.11 and F11.21, which correspond to opioid abuse or dependence in remission (see Supplementary Table S1). These codes have been previously used to effectively detect illicit substance use as a behavior.^8^ Based on demographic reporting, we categorized sex (male and female) and race/ethnicity (White and non-White). The group of non-White includes those who identified their race/ethnicity group as Black, Hispanic, and Other (individuals who do not identify as White, Black, or Hispanic). Race/ethnicity was dichotomized due to the relatively small sample size of the non-White group.

For each extracted patient, we used the ICD-10 Procedure Coding System codes for claims classification to identify their sequence of treatment utilization (see Supplementary Table S2). Procedure codes were aggregated into four categories (1) detoxification, (2) psychosocial services that include individual or group counseling and psychotherapy, (3) medication-assisted treatment that includes agonist medication (methadone or buprenorphine) or antagonist medication (naltrexone), and (4) comprehensive treatment that includes a combination of agonist or antagonist meditation with one type of psychosocial service.

We verified the significance on difference in the proportion of patients receiving treatment between population subgroups using a two-proportion *Z*-test. All statistical tests were two-sided, and *p*<0.05 was used for statistical significance.

## Study Results

Over the study period, there were 52,994 Indiana Medicaid enrollees diagnosed with OUD. The demographic distribution for the study population, consisted of 29,925 (56.47%) female and 23,069 (43.53%) male, 47,110 (91.06%) White, and 4627 (8.94%) non-White (Table 1). Note that the totals for race/ethnicity were slightly less than for sex since some enrollees did not report race/ethnicity. In addition, 5.41% of enrollees with an OUD received at least one category of treatment procedure (detoxification, psychosocial, MAT, or comprehensive), with detoxification being the most likely treatment.

**Table 1.** Indiana Medicaid Enrollees Who Received Treatment for Opioid Use Disorder in 2018–2019

	Detoxification		Psychosocial services						Medication-assisted treatment				Comprehensive treatment			
			Individual counseling		Group counseling		Psychotherapy		Agonist medication		Antagonist medication		Antagonist+psychosocial		Agonist+psychosocial	
	*N* (%)	s.e.	*N* (%)	s.e.	*N* (%)	s.e.	*N* (%)	s.e.	*N* (%)	s.e.	*N* (%)	s.e.	*N* (%)	s.e.	*N* (%)	s.e.
Total (*N*=52,994)	2781 (5.25%)	0.002	30 (0.05%)	0	414 (0.78%)	0.001	2 (0.00%)	0	271 (0.51%)	0.001	245 (0.46%)	0	10 (0.01%)	0	28 (0.05%)	0
Male (*N*=23,069)	1428 (6.19%)	0.002	10 (0.04%)	0	211 (0.92%)	0.001	1 (0.00%)	0	134 (0.58%)	0.001	122 (0.53%)	0	6 (0.02%)	0	14 (0.06%)	0
Female (*N*=29,925)	1353 (4.52%)	0.001	12 (0.04%)	0	203 (0.68%)	0	1 (0.00%)	0	137 (0.45%)	0	123 (0.41%)	0	4 (0.01%)	0	14 (0.04%)	0
Men vs. Women (*p*-value)	*p*=0.000		*p*=0.500		*p*=0.000		^***^		*p*=0.016		*p*=0.042		^***^		*p*=0.40	

	Detoxification			Psychosocial services			Agonist medication			Antagonist medication			Comprehensive treatment			
	*N* (%)		s.e.	*N* (%)		s.e.	*N* (%)		s.e.	*N* (%)		s.e.	*N* (%)		s.e.	
Total (*N*=51,737)	2742 (5.30%)		0.002	430 (0.83%)		0	268 (0.51%)		0	240 (0.46%)		0	31 (0.06%)		0	
White (*N*=47,110)	2557 (5.42%)		0.001	403 (0.86%)		0	233 (0.49%)		0	217 (0.46%)		0	30 (0.06%)		0	
Non-White (*N*=4,627)	191 (4.12%)		0.002	27 (0.58%)		0	35 (0.75%)		0	24 (0.51%)		0	1 (0.02%)		0	
White vs. Non-White (*p*-value)	*p*=0.000			*p*=0.000			*p*=0.002			*p*=0.320			^***^			

^***^ , not possible to compare due to sample size.

s.e., standard error of the proportion.

Across all groups, group counseling was the most common type of psychosocial service compared with individual counseling and psychotherapy. Across treatment categories, men were 37% more likely to receive detoxification services, 35% more likely to receive group counseling, 28% more likely to receive agonist medication, and 26% more likely to receive antagonist medication, compared with women. However, there was no statistically significant difference in the proportion of men receiving comprehensive treatment compared with women.

Furthermore, White enrollees with an OUD were 32% more likely to receive detoxification services and 46% more likely to receive psychosocial services compared with non-White enrollees. Regarding agonist medication, non-White enrollees were 53% more likely to receive treatment than White enrollees. However, this primarily came from differences in methadone treatment. For those receiving agonists, non-White enrollees were >22 times more likely than White enrollees to receive methadone, whereas White enrollees were 1.18 times more likely to receive buprenorphine compared with non-White enrollees (data not shown).

These results are consistent with the findings from Goedel et al, which reported that the U.S. counties with predominant non-White communities had more access to methadone treatment, whereas counties with predominantly White communities had more facilities providing buprenorphine treatment.^9^ There was no significant difference between non-White and White enrollees in the other treatment categories. However, due to the small sample size of non-Whites receiving treatment, the comparison could not be performed in all categories.

## Discussion

Although Medicaid began covering treatment services for enrollees with OUD in Indiana at the start of 2018, very few such patients received these services. Furthermore, treatment varied by sex and race/ethnicity. The number of women diagnosed with OUD constituted 56% of the study population in Indiana. Despite their increased incidence, women were less likely than men to enter MAT programs. Providing childcare and domestic counseling through woman-centered services could potentially help reduce these barriers to treatment access.^10^

Detoxification was the most likely treatment received. There are multiple possible reasons. First, providers are required to have a DEA DATA 2000 waiver to prescribe buprenorphine for OUD and, therefore, not all providers are able to prescribe this medication. Unless the patient is seen in a clinic with integrated behavioral health, providers must know where to refer patients for psychotherapy and counseling services and those services must exist in the community. Second, detoxification is the abstinence only approach to treating opioid use, and this finding is not surprising in the context of provider stigma related to opioid use.^11^

This study has several limitations. First, we did not take factors impacting the access to addiction treatment into account, for example, the distance a patient traveled to receive treatment, car ownership, or the location and availability of providers. Second, as methadone has not always been covered by Indiana Medicaid, we could only collect utilization data for methadone from January 2018 to March 2019. Given the small cohort size, we were not able to break down our comparative analysis at the level of specific pharmacotherapy options for sufficient statistical significance. In addition, we would caution against generalizing the differences in antagonist and agonist medication prescriptions to the general population from the Indiana Medicaid enrollees. Finally, it is possible that individuals could have paid for treatment out of pocket or through reimbursed care in which case a Medicaid claim would not be filed.

Given the national concern on health care disparities among race/ethnicity and sex groups, the need for conducting a detailed and comprehensive examination of treatment patterns is pressing. Access to health care, cultural and language differences, communication, and trust barriers are contributors to these disparities.^12^ This study found that only a small percentage of people diagnosed with an OUD or medically documented opioid use received treatment. There were also race/ethnicity and sex disparities in the type of treatment provided. Although the data in this study do not allow for the determination of the source of these disparities, it does demonstrate the need for further study into the underlying reasons for low treatment rates and disparities. Some previous studies have found that stereotyping or decisional biases by clinicians in some situations may play a role.^13–15^

Encouraging primary care providers to use evidence-based assessment procedures may help them more accurately assess OUD risks and also help mitigate potential biases in treatment and referral.^13,15^ Furthermore, education on current treatment differences based on sex or race/ethnicity could play an important role.

## Supplementary Material

Supplemental data

Supplemental data

Supplemental data

## Abbreviations Used

MAT: medication-assisted treatment
OUD: opioid use disorder
