# Supplementary material for: Opioid-Related Treatment Disparities Among Medicaid Enrollees in Indiana
Source: Health Equity. 2023 Feb 1;7(1):76–9. doi: 10.1089/heq.2021.0154 (PMC9982140; doi:10.1089/heq.2021.0154)
Supplement: Supplemental data [file Suppl_FigS1.docx]

**Figure S1. Study population**
