# Supplementary material for: Opioid-Related Treatment Disparities Among Medicaid Enrollees in Indiana
Source: Health Equity. 2023 Feb 1;7(1):76–9. doi: 10.1089/heq.2021.0154 (PMC9982140; doi:10.1089/heq.2021.0154)
Supplement: Supplemental data [file Suppl_TableS1.docx]

**Table A1. ICD-10 codes used to identify study population**

|  | **Opioid abuse, use and dependence** |
| --- | --- |
| **Code** | **Description** |
| F1110 | Opioid abuse, uncomplicated |
| F11120 | Opioid abuse with intoxication, uncomplicated |
| F11121 | Opioid abuse with intoxication delirium |
| F11122 | Opioid abuse with intoxication with perceptual disturbance |
| F11129 | Opioid abuse with intoxication, unspecified |
| F1114 | Opioid abuse with opioid-induced mood disorder |
| F11150 | Opioid abuse with opioid-induced psychotic disorder with delusions |
| F11151 | Opioid abuse with opioid-induced psychotic disorder with hallucinations |
| F11159 | Opioid abuse with opioid-induced psychotic disorder, unspecified |
| F11181 | Opioid abuse with opioid-induced sexual dysfunction |
| F11182 | Opioid abuse with opioid-induced sleep disorder |
| F11188 | Opioid abuse with other opioid-induced disorder |
| F1119 | Opioid abuse with unspecified opioid-induced disorder |
| F1120 | Opioid dependence, uncomplicated |
| F11220 | Opioid dependence with intoxication, uncomplicated |
| F11221 | Opioid dependence with intoxication delirium |
| F11222 | Opioid dependence with intoxication with perceptual disturbance |
| F11229 | Opioid dependence with intoxication, unspecified |
| F1123 | Opioid dependence with withdrawal |
| F1124 | Opioid dependence with opioid-induced mood disorder |
| F11250 | Opioid dependence with opioid-induced psychotic disorder with delusions |
| F11251 | Opioid dependence with opioid-induced psychotic disorder with hallucinations |
| F11259 | Opioid dependence with opioid-induced psychotic disorder, unspecified |
| F11281 | Opioid dependence with opioid-induced sexual dysfunction |
| F11282 | Opioid dependence with opioid-induced sleep disorder |
| F11288 | Opioid dependence with other opioid-induced disorder |
| F1129 | Opioid dependence with unspecified opioid-induced disorder |
| F1190 | Opioid use, unspecified, uncomplicated |
| F11920 | Opioid use, unspecified with intoxication, uncomplicated |
| F11921 | Opioid use, unspecified with intoxication delirium |
| F11922 | Opioid use, unspecified with intoxication with perceptual disturbance |
| F11929 | Opioid use, unspecified with intoxication, unspecified |
| F1193 | Opioid use, unspecified with withdrawal |
| F1194 | Opioid use, unspecified with opioid-induced mood disorder |
| F11950 | Opioid use, unspecified with opioid-induced psychotic disorder with delusions |
| F11951 | Opioid use, unspecified with opioid-induced psychotic disorder with hallucinations |
| F11959 | Opioid use, unspecified with opioid-induced psychotic disorder, unspecified |
| F11981 | Opioid use, unspecified with opioid-induced sexual dysfunction |
| F11982 | Opioid use, unspecified with opioid-induced sleep disorder |
| F11988 | Opioid use, unspecified with other opioid-induced disorder |
| F1199 | Opioid use, unspecified with unspecified opioid-induced disorder |
| **ICD10** | **Poisoning** |
| **Code** | **Description** |
| T400X1A | Poisoning by opium, accidental (unintentional), initial encounter |
| T400X1D | Poisoning by opium, accidental (unintentional), subsequent encounter |
| T400X1S | Poisoning by opium, accidental (unintentional), sequela |
| T400X2A | Poisoning by opium, intentional self-harm, initial encounter |
| T400X2D | Poisoning by opium, intentional self-harm, subsequent encounter |
| T400X2S | Poisoning by opium, intentional self-harm, sequela |
| T400X3A | Poisoning by opium, assault, initial encounter |
| T400X3D | Poisoning by opium, assault, subsequent encounter |
| T400X3S | Poisoning by opium, assault, sequela |
| T400X4A | Poisoning by opium, undetermined, initial encounter |
| T400X4D | Poisoning by opium, undetermined, subsequent encounter |
| T400X4S | Poisoning by opium, undetermined, sequela |
| T401X1A | Poisoning by heroin, accidental (unintentional), initial encounter |
| T401X1D | Poisoning by heroin, accidental (unintentional), subsequent encounter |
| T401X1S | Poisoning by heroin, accidental (unintentional), sequela |
| T401X2A | Poisoning by heroin, intentional self-harm, initial encounter |
| T401X2D | Poisoning by heroin, intentional self-harm, subsequent encounter |
| T401X2S | Poisoning by heroin, intentional self-harm, sequela |
| T401X3A | Poisoning by heroin, assault, initial encounter |
| T401X3D | Poisoning by heroin, assault, subsequent encounter |
| T401X4A | Poisoning by heroin, undetermined, initial encounter |
| T401X4D | Poisoning by heroin, undetermined, subsequent encounter |
| T401X4S | Poisoning by heroin, undetermined, sequela |
| T401X3S | Poisoning by heroin, assault, sequela |
| T402X1A | Poisoning by other opioids, accidental (unintentional)initial encounter |
| T402X1D | Poisoning by other opioids, accidental (unintentional)subsequent encounter |
| T402X1S | Poisoning by other opioids, accidental (unintentional), sequela |
| T402X2A | Poisoning by other opioids, intentional self-harm, initial encounter |
| T402X2D | Poisoning by other opioids, intentional self-harm, subsequent encounter |
| T402X2S | Poisoning by other opioids, intentional self-harm, sequela |
| T402X3A | Poisoning by other opioids, assault, initial encounter |
| T402X3D | Poisoning by other opioids, assault, subsequent encounter |
| T402X3S | Poisoning by other opioids, assault, sequela |
| T402X4A | Poisoning by other opioids, undetermined, initial encounter |
| T402X4D | Poisoning by other opioids, undetermined, subsequent encounter |
| T402X4S | Poisoning by other opioids, undetermined, sequela |
| T403X1A | Poisoning by methadone, accidental (unintentional)initial encounter |
| T403X1D | Poisoning by methadone, accidental (unintentional)subsequent encounter |
| T403X1S | Poisoning by methadone, accidental (unintentional), sequela |
| T403X2A | Poisoning by methadone, intentional self-harm, initial encounter |
| T403X2D | Poisoning by methadone, intentional self-harm, subsequent encounter |
| T403X2S | Poisoning by other opioids, intentional self-harm, sequela |
| T403X3A | Poisoning by methadone, assault, initial encounter |
| T403X3D | Poisoning by methadone, assault, subsequent encounter |
| T403X3S | Poisoning by methadone, assault, sequela |
| T403X4A | Poisoning by methadone, undetermined, initial encounter |
| T403X4D | Poisoning by methadone, undetermined, subsequent encounter |
| T403X4S | Poisoning by methadone, undetermined, sequela |
