# Supplementary material for: Opioid-Related Treatment Disparities Among Medicaid Enrollees in Indiana
Source: Health Equity. 2023 Feb 1;7(1):76–9. doi: 10.1089/heq.2021.0154 (PMC9982140; doi:10.1089/heq.2021.0154)
Supplement: Supplemental data [file Suppl_TableS2.docx]

**Table A2. ICD10-PCS codes to identify treatment received by the study population**

| **Individual substance abuse treatment - counseling** | |
| --- | --- |
| **Code** | **Description** |
| HZ2ZZZZ | Substance abuse treatment detoxification services |
| HZ30ZZZ | Substance abuse treatment individual counseling |
| HZ31ZZZ | Substance abuse treatment individual counseling, behavioral |
| HZ32ZZZ | Substance abuse treatment individual counseling, cognitive |
| HZ33ZZZ | Substance abuse treatment individual counseling, 12-step |
| HZ34ZZZ | Substance abuse treatment individual counseling, interpersonal |
| HZ35ZZZ | Substance abuse treatment individual counseling, vocational |
| HZ36ZZZ | Substance abuse treatment individual counseling, psychoeducation |
| HZ37ZZZ | Substance abuse treatment individual counseling, motivational enhancement |
| HZ38ZZZ | Substance abuse treatment individual counseling, confrontational |
| HZ39ZZZ | Substance abuse treatment individual counseling, continuing care |
| HZ3BZZZ | Substance abuse treatment individual counseling, spiritual |
| HZ3CZZZ | Substance abuse treatment individual counseling, pre/post-test infectious disease |
| **Group substance abuse treatment - counseling** | |
| **Code** | **Description** |
| HZ40ZZZ | Substance abuse treatment group counseling |
| HZ41ZZZ | Substance abuse treatment group counseling, behavioral |
| HZ42ZZZ | Substance abuse treatment group counseling, cognitive-behavioral |
| HZ43ZZZ | Substance abuse treatment group counseling, 12-step |
| HZ44ZZZ | Substance abuse treatment group counseling, interpersonal |
| HZ45ZZZ | Substance abuse treatment group counseling, vocational |
| HZ46ZZZ | Substance abuse treatment group counseling, psychoeducation |
| HZ47ZZZ | Substance abuse treatment group counseling, motivational enhancement |
| HZ48ZZZ | Substance abuse treatment group counseling, confrontational |
| HZ49ZZZ | Substance abuse treatment group counseling, continuing care |
| HZ4BZZZ | Substance abuse treatment group counseling, spiritual |
| HZ4CZZZ | Substance abuse treatment group counseling, pre/post-test infectious disease |
| **Individual substance abuse treatment - psychotherapy** | |
| **Code** | **Description** |
| HZ50ZZZ | Substance abuse treatment individual psychotherapy |
| HZ51ZZZ | Substance abuse treatment individual psychotherapy, behavioral |
| HZ52ZZZ | Substance abuse treatment individual psychotherapy, cognitive |
| HZ53ZZZ | Substance abuse treatment individual psychotherapy, 12-step |
| HZ54ZZZ | Substance abuse treatment individual psychotherapy, interpersonal |
| HZ55ZZZ | Substance abuse treatment individual psychotherapy, interactive |
| HZ56ZZZ | Substance abuse treatment individual psychotherapy, psychoeducation |
| HZ57ZZZ | Substance abuse treatment individual psychotherapy, motivational enhancement |
| HZ58ZZZ | Substance abuse treatment individual psychotherapy, confrontational |
| HZ59ZZZ | Substance abuse treatment individual psychotherapy, supportive |
| HZ5BZZZ | Substance abuse treatment individual psychotherapy, psychoanalysis |
| HZ5CZZZ | Substance abuse treatment individual psychotherapy, psychodynamic |
| HZ5DZZZ | Substance abuse treatment individual psychotherapy, psychophysiological |
| HZ63ZZZ | Substance abuse treatment family counseling |
| **Substance abuse treatment - medication management** | |
| **Code** | **Description** |
| HZ80ZZZ | Substance abuse treatment medication management |
| HZ81ZZZ | Substance abuse treatment medication management, methadone maintenance |
| HZ82ZZZ | Substance abuse treatment medication management, levo-alpha-acetylmethadol (LAAM) |
| HZ83ZZZ | Substance abuse treatment medication management, Antabuse |
| HZ84ZZZ | Substance abuse treatment medication management, naltrexone |
| HZ85ZZZ | Substance abuse treatment medication management, naloxone |
| HZ86ZZZ | Substance abuse treatment medication management, clonidine |
| HZ87ZZZ | Substance abuse treatment medication management, bupropion |
| HZ88ZZZ | Substance abuse treatment medication management, psychiatric medication |
| HZ89ZZZ | Substance abuse treatment medication management, other replacement medication |
| **Substance abuse treatment medication - pharmacotherapy** | |
| **Code** | **Description** |
| HZ90ZZZ | Substance abuse treatment medication pharmacotherapy |
| HZ91ZZZ | Substance abuse treatment medication pharmacotherapy, methadone maintenance |
| HZ92ZZZ | Substance abuse treatment medication pharmacotherapy, levo-alpha-acetylmethadol (LAAM) |
| HZ93ZZZ | Substance abuse treatment medication pharmacotherapy, Antabuse |
| HZ94ZZZ | Substance abuse treatment medication pharmacotherapy, naltrexone |
| HZ95ZZZ | Substance abuse treatment medication pharmacotherapy, naloxone |
| HZ96ZZZ | Substance abuse treatment medication pharmacotherapy, clonidine |
| HZ97ZZZ | Substance abuse treatment medication pharmacotherapy, bupropion |
| HZ98ZZZ | Substance abuse treatment medication pharmacotherapy, psychiatric medication |
| HZ99ZZZ | Substance abuse treatment medication pharmacotherapy, other replacement medication |
